# Posterior Inferior Cerebellar Artery Stroke Due to a Severe Right Vertebral Artery Stenosis With a Left Cervical Internal Carotid Artery Dissection: What’s Next?

**DOI:** 10.7759/cureus.55598

**Published:** 2024-03-05

**Authors:** Sam Kara, Fredy G Gutierrez Munoz, Jeremy Eckes, Sahar S Abdelmoneim, Kester Nedd

**Affiliations:** 1 Department of Neurology, Larkin Community Hospital Palm Springs Campus, Hialeah, USA; 2 Department of Internal Medicine, Larkin Community Hospital Palm Springs Campus, Hialeah, USA

**Keywords:** prevention of ischemic stroke, acute ischemic stroke (ais), vertebral artery stenosis, carotid artery dissection, stroke management

## Abstract

Guidelines for the treatment and management of ischemic strokes triggered by stenosis versus dissection are well established. However, the presence of both entities in the same patient, although rare, poses challenges for short- and long-term treatment. Here, we describe the case of a 55-year-old man who presented to the emergency department with a 72-hour history of headache, dizziness, unbalanced gait, nausea, and two episodes of vomiting. Stroke was initially suspected, but the computerized tomography (CT) scan showed no hemorrhage. His magnetic resonance imaging (MRI) showed right inferior cerebellar acute ischemia in the territory of the right posterior inferior cerebellar artery (PICA), with smaller foci of early acute infarcts in the bilateral inferior cerebellum. Furthermore, magnetic resonance angiography (MRA) and CT angiography revealed right vertebral artery stenosis and left cervical internal carotid artery dissection (ICAD). This clinical report describes a rare case of stroke secondary to vertebral artery stenosis with concomitant carotid artery dissection. The treatment course and evolution are presented.

## Introduction

Ischemic stroke (IS), defined as an interruption of blood supply to the brain, is the most common type of stroke, with approximately 690,000 adults in the United States thought to experience ischemic stroke annually [1]. In ischemia, neurons receive afferent input from other neurons; when this afferent input is lost from an ischemic stroke, these neurons become silent, which clinically manifests as loss of function. However, when the afferent inputs are restored, these silent neurons gradually restore and regain function. This concept is also known as "diaschisis" [1].

IS may improve or worsen over time and can be fatal, although approximately 87% of people who experience IS survive. However, most stroke survivors suffer handicaps and require physical therapy [2].

Ischemia causes brain abnormalities that can be visualized by brain computed tomography (CT) or magnetic resonance imaging (MRI). Ischemic changes can also be detected on diffusion-weighted imaging (DWI). Additionally, blood vessel abnormalities are identified by magnetic resonance angiography (MRA) or CT angiography. These radiographic modalities are used to follow up patients after treatment to reduce the risk of treatment complications such as bleeding [3]. Thus, understanding and identifying risk factors is imperative. Development of stroke risk metrics can be used to quantify the risk over time after the initial stroke. Risk factors known to lead to IS include heart disease, blood clotting problems, and blood vessel abnormalities such as those caused by hypertension, diabetes, high cholesterol, and smoking. These risk factors affect both short- and long-term treatment of IS. The ABCD2 clinical risk prediction score is a popular metric used to assess risks based on the metric score and optimize treatment to decrease the risk of recurrent stroke over time [4].

A common cause of IS before the age of 45 is dissection of the cervical arteries (carotid and vertebral arteries). Carotid artery dissection (CAD) is the underlying etiology of stroke in approximately 2.5% of all stroke cases and 5% to 25% in individuals aged 30-45 years. CAD has a mortality rate of up to 5%, and after three months of treatment and evolution, 90% of cases achieve full luminal permeability [5,6].

Treatment for IS can be classified as acute management or secondary prevention. During acute treatment, the primary objective is to restore blood flow to brain tissue that is at risk. Specific strategies may be used to treat IS caused by other conditions, such as thrombolysis with tissue-type plasminogen activators and, under certain circumstances, endovascular therapies. For the prevention of early and late stroke recurrences, anticoagulants and antiplatelet agents are generally used. When applicable, secondary stroke prevention recommendations should be followed with regard to other causes of IS [4,7].

Here, we present and discuss the case of a male patient in terms of the management of a concomitant cerebellar stroke and internal carotid artery dissection (ICAD).

## Case presentation

A 55-year-old, right-handed male with a medical history of untreated hypertension and chronic cervical pain, for which he received weekly chiropractic manipulations over an eight-month period (the last visit was a week before presentation), also had an alcohol use disorder, consuming 21 bottles of 12 fl oz beer per week. The patient presented to the emergency department with complaints of an unremitting headache, dizziness, unbalanced gait, nausea, and two episodes of vomiting, all of which had been present for a 72-hour period. The patient had never smoked and does not take any home medications. The headache was located in the posterior occiput and radiated anteriorly. The headaches coincided with physical exercise and alcohol and were not relieved by aspirin or ibuprofen. The patient denied having any fever, chills, lightheadedness, neck stiffness, numbness, slurred speech, loss of consciousness, blurry vision, changes in hearing, seizures, muscle weakness, bowel or urine incontinence, abdominal pain, flank pain, nausea or vomiting, or any prior similar symptoms. The patient’s family history is unremarkable, and his body mass index (BMI) was 28.3 kg/m².

**Figure 1:**
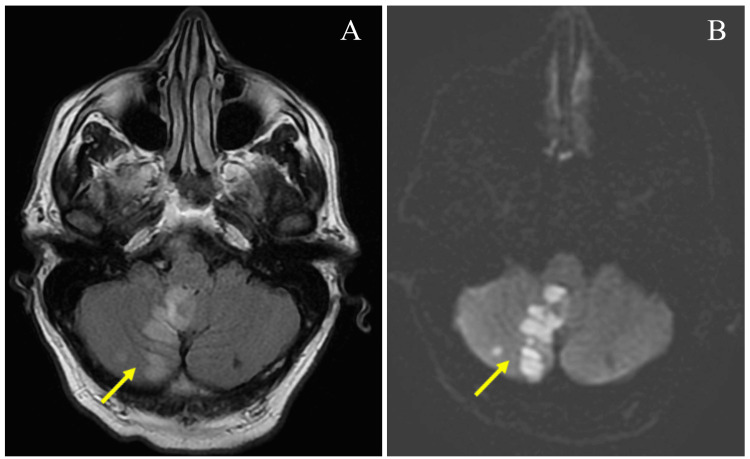
Infarction of right cerebellar hemisphere Brain magnetic resonance imaging (A: T2-Flair and B: T2-Diffusion Weighted Imaging) showing a hyperintense lesion (arrow) in the right inferior cerebellar hemisphere representing an acute confluent infarction with a small hemorrhagic conversion from right posterior inferior cerebellar artery (PICA) territory stroke. Additional smaller foci of early acute versus subacute infarcts in the bilateral inferior cerebellum are noted.

**Figure 2:**
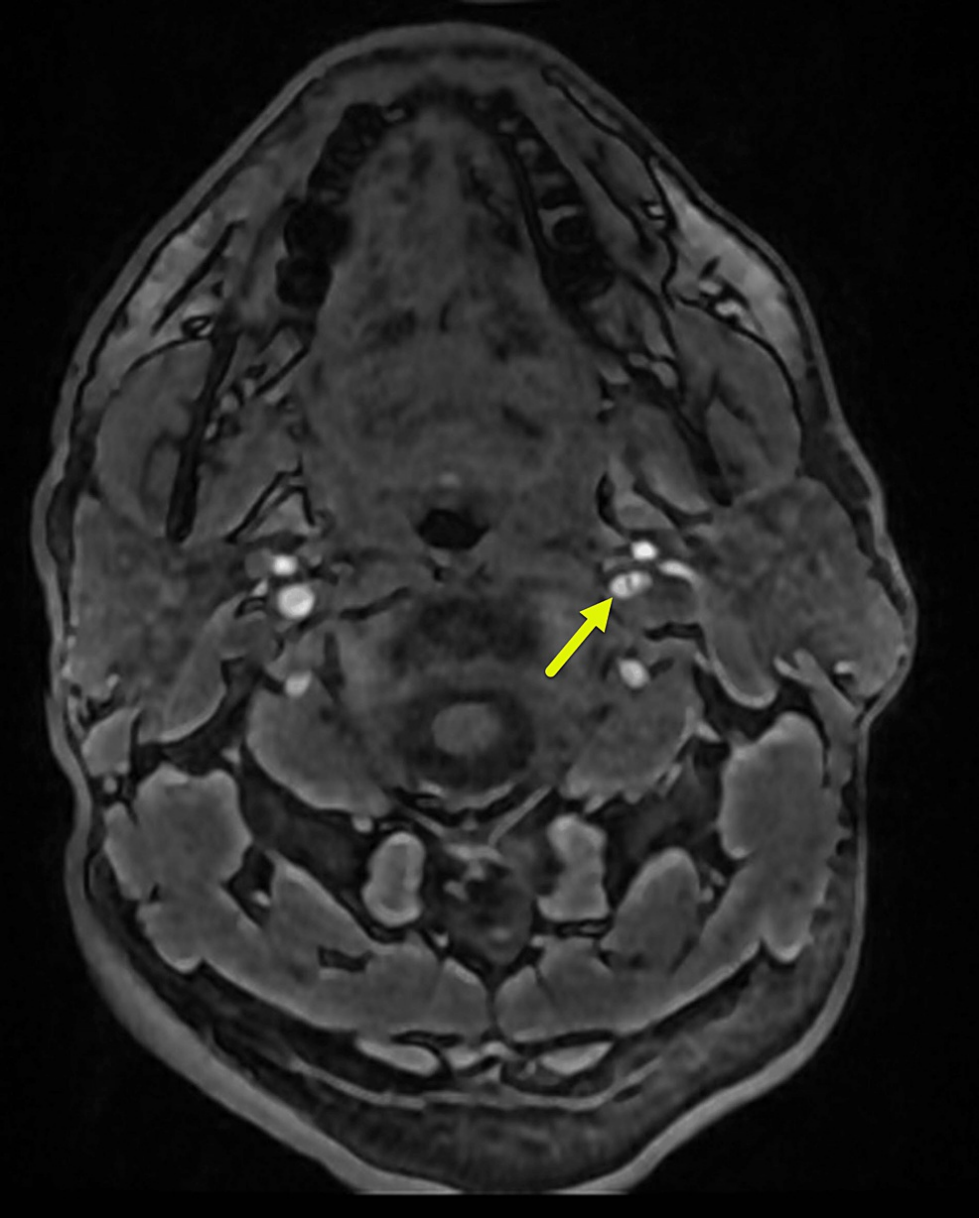
Left cervical internal carotid artery (ICA) dissection Brain contrast magnetic resonance angiography showing a linear filling defect, creating a double lumen (yellow arrow) visible in the left cervical internal carotid artery (ICA), a finding of ICA dissection versus thrombus. The remaining portions of the left internal carotid artery and the right internal carotid arteries are patent without evidence of significant stenosis or occlusion.

**Figure 3:**
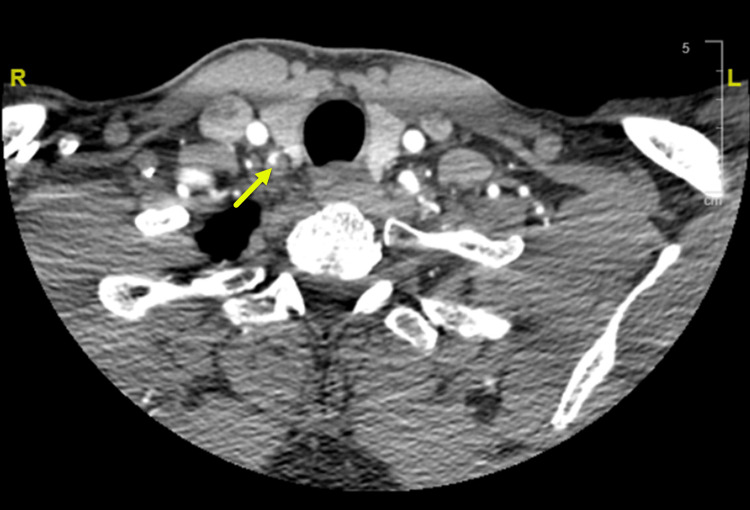
Right vertebral artery stenosis CT angiogram (CTA) of head and neck with contrast showing the circumferential noncalcified plaque of the V1 segment proximal right vertebral artery causing severe, greater than 90% stenosis. The left vertebral artery is patent. The vertebral arteries are symmetric in size, both contribute to the basilar artery.

The patient’s vital signs upon arrival showed the following: blood pressure, systolic of 176 mmHg and diastolic of 93 mmHg; oxygen saturation, 96% at atmospheric air; body temperature, 98.3ºF; and heart rate, 47 beats/min. Physical examination showed him alert and oriented in time, person, place, and event, with normal speech, intact external ocular motion with noted horizontal nystagmus, pupils that were equal, rounded, and reactive to light and accommodation, a non-tender neck, no audible carotid bruit, no masses, and full range of motion intact. Neurologic examination showed a Glasgow Coma Scale of 15/15, intact cranial nerves II-XII, equal bilateral reflexes, no sensory or motor deficits, and normal gait. Head Impulse, Nystagmus, and Test of Skew (HINTS) examination revealed right side horizontal nystagmus without vertical nystagmus. Truncal ataxia was noted upon ataxia assessment. The National Institutes of Health Stroke Scale (NIHSS) was 0. Heart and lung examinations were normal. Chest X-ray and electrocardiogram on admission were unremarkable. The patient’s baseline laboratory testing, including complete blood counts, coagulation profile, troponin, thyroid-stimulating hormone (TSH), and basic metabolic panel: glucose, blood urea nitrogen (BUN), creatinine, carbon dioxide, chloride, potassium, sodium, and calcium, were unremarkable. The COVID rapid test was negative. CT angiography of the head and neck showed right cerebellar hypodensity corresponding to the infarct observed on the brain MRI of the same day (Figure 1), with no acute hemorrhage transformation; right vertebral artery V1 segment high grade; more than 90% stenosis from circumferential noncalcified plaque (The North American Symptomatic Carotid Endarterectomy Trial (NASCET) criteria); and proximal left cervical internal carotid artery with a linear filling defect and thrombus versus dissection (Figures 2, 3). No acute ischemic event of the anterior vascular territories (middle and anterior cerebral arteries) resulted as a consequence of the carotid dissection.

The neurological Interventional Radiology team was activated, and a loading dose of dual antiplatelet therapy (DAPT), including aspirin (325 mg) and clopidogrel (300 mg) was administered, followed by daily maintenance doses of 81 mg and 75 mg, respectively, as well as a high dose of atorvastatin (80 mg) daily. The patient underwent digital subtraction cerebral angiography, which showed right vertebral artery stenosis of more than 90%. The hospital course was uncomplicated, and the patient was hemodynamically stable. He was instructed to continue with antihypertensive medications (telmisartan/amlodipine 40/5 mg daily), high-intensity atorvastatin 80 mg daily, dual antiplatelet aspirin (81 mg) and clopidogrel (75 mg) daily for six months, and aspirin for life. The patient was instructed not to attend chiropractic sessions and to follow up with primary care physicians for cardiovascular risk factor optimizations. Hypercoagulation workup showed elevated lupus antibody levels, and the patient was referred to hematology for further evaluation, the results of which were negative (Table 1).

**Table 1:** Hypercoagulable workup Lupus anticoagulant was detected. Prolonged PT reasons including medication side effects (anticoagulant during hospitalization). Other rare factors include factor deficiencies and weak inhibitors. PT: prothrombin time. PTT: partial thromboplastin time.

Test	Value	Unit	Reference
Lupus anticoagulant PT	13.1	Seconds	9.4-13.5
Lupus anticoagulant mix PT Pat/Norm	12.1	Seconds	9.4-12.5
Lupus anticoagulant PTT	32.5	Seconds	25.1-36.5
Protein C activity	87	%	70-140
Activated protein C resistance	2.85	Ratio	>2.24 (Negative)
Protein S activity	98	%	63.5-149

MRI and MRA at the three-month follow-up showed no new IS or hemorrhagic events with the current treatment plan, and his carotid artery dissection remained stable compared to the initial MRA (Figure 2). Clinically, he recovered completely, with no neurological deficits. 

## Discussion

Our unusual case debuted simultaneous left cervical ICAD and right cerebellar infarct due to occlusion of the right posterior inferior cerebellar artery (PICA) branch of the vertebral artery, which, in its V1 segment, showed more than 90% of stenosis secondary to a circumferential non-calcified plaque. The patient received chiropractic manipulation a few weeks prior to ICAD. We consider that his carotid dissection was likely related to that session. Despite the lack of direct evidence to support chiropractic manipulation's association with CAD, clinical reports have suggested that mechanical forces could play a role in cervical and vertebral artery dissection in young stroke patients [7].

The patient’s acute ischemic infarction was due to atherosclerotic arteriogenic embolism, and the patient was referred for an urgent MRI, which showed a right cerebellar hemispheric infarct. The physiopathology behind the IS is possibly an artery-to-artery micro-emboli secondary to vertebral artery atheromatous stenosis, and the hypertensive crisis. Several studies have suggested that thromboembolism is the main mechanism of stroke in ICAD, rather than the hemodynamic compromise per se [8,9]; however, in our case, it was not linked given the unrelated vascular anatomy distribution.

Patients with acute IS related to CAD without contraindications should be treated with thrombolysis without delay within 4.5 hours. The use of tissue-type plasminogen activator (TPA) is safe for patients with acute IS due to CAD, as it does not increase the risk of intracranial hemorrhage [7,10]. TPA was not used in our case due to the patient’s late arrival at the hospital (72 hours after the onset of symptoms).

For secondary stroke prevention in patients with transient ischemic attack (TIA) or IS after vertebral arterial or extracranial carotid dissection, antiplatelet or anticoagulant therapy for three to six months is reasonable to prevent recurrent events [4]. Previous non-randomized studies [11,12] did not find any significant distinctions between the two therapies until the randomized cervical artery dissection in stroke study (CADISS) trial [13], which did not demonstrate disparities in stroke prevention, residual stenosis, or occlusion between patients treated with antiplatelets or anticoagulants following CAD. Moreover, the risk of stroke recurrence was low for up to one year. The Treat-CAD study [14] did not find non-inferiority with the use of aspirin over the use of anticoagulants in the context of ICAD. However, this study was limited by the small sample size and the greater number of side effects associated with the use of vitamin K antagonists. In addition, this study did not compare the use of DAPT versus anticoagulants in this context. Following the CADISS trial findings and considering the serious side effects of anticoagulants, such as bleeding risk, we decided to use DAPT for six months.

Similarly, a systematic review and meta-analysis involving 2064 patients that compared the outcomes of antiplatelet therapy versus anticoagulation in cervical artery dissection revealed no significant difference between the two groups, except for primary ischemic stroke (IS) (randomized controlled trials analyzed) and complete recanalization (observational studies analyzed), which indicated a significant favor for anticoagulants over antiplatelets. Although primary IS is an important outcome, it is important to consider several other factors that may affect these results; these include incomplete adjustment for the confounding effects of antiplatelet-anticoagulant doses, frequency, administration compliance, and others. Well-designed studies are recommended to determine whether unnecessary anticoagulation can be avoided in CAD [15].

Currently, there is no consensus on the optimal duration for either therapy. Antithrombotic therapy is usually continued for up to six months based on the natural progression of ICAD and the likelihood of symptom recurrence if anticoagulant treatment is discontinued within the first three to six months following dissection onset but is rare beyond this timeframe [4,6]. There have been reports recommending the use of DAPT over a short period of time (21-90 days) with aspirin and clopidogrel in patients with non-cardioembolic IS or TIA [16,17]. Meta-analyses have demonstrated a reduction in recurrent stroke from DAPT compared to aspirin monotherapy, but the benefit is limited if not initiated early (<7 days) after the index event. While most studies have demonstrated an increase in bleeding risk following DAPT, this was offset by the stroke prevention benefit if DAPT was limited to short-term use [4,18].

Surgical or endovascular procedures are reserved only for patients who experience definite recurrent cerebral ischemic events while on appropriate antithrombotic therapy; in patients with absolute contraindications for anticoagulation or antiplatelet treatment; in cases where carotid aneurysms and/or tight carotid stenosis persisting or developing de novo; iatrogenic dissections occurring during intravascular procedures; or patients with hemodynamic hypoperfusion (involvement of multiple vessels or poor collateral vessels) or pseudoaneurysm formation. Such procedures are technically demanding and are associated with high morbidity and periprocedural risks, which narrows the selection of patient candidates for these therapies [6,19]. In general, the natural history of ICAD would follow in persistent or transient stenosis or occlusion, segued by recanalization and return to normal blood flow within three to six months of treatment. Transient ICA stenosis or occlusion is associated with a 0.9% annual stroke risk, whereas permanent ICA stenosis or occlusion carries a 2.1% annual stroke risk [6,20]. Based on these percentages involving the long-term outcomes associated with ICAD and the current literature, conservative management with either antiplatelet or anticoagulant therapy should be the standard of care for patients with ICAD.

## Conclusions

In this report, we present an unusual case of PICA stroke with > 90% right vertebral artery stenosis and concomitant left ICA dissection. Our case further illustrates the potential complications associated with chiropractic manipulation. The patient presented with an NIHSS of 0, and the MRI findings were positive for acute ischemic stroke. His treatment plan consisted of high-intensity atorvastatin (80 mg daily) and DAPT for six months followed by aspirin for life. The need to limit his systolic blood pressure to <140 and cease any activity that would lead to increased blood pressure or may cause trauma to the head to limit intracerebral bleeding incidents was emphasized. The workup for any hypercoagulable state was negative. Follow-up brain MRA revealed that the carotid dissection was similar to the initial MRA. This patient recovered completely with no focal neurological deficits following conservative management without any medical or radiologic complications, demonstrating the long-term benefits of using this approach for patients with concurrent acute ischemic stroke and ICAD.

## References

[1] Donkor ES (2018). Stroke in the 21(st) century: a snapshot of the burden, epidemiology, and quality of life. Stroke Res Treat.

[2] Chuluunbaatar E, Chou YJ, Pu C (2016). Quality of life of stroke survivors and their informal caregivers: a prospective study. Disabil Health J.

[3] Smith AG, Rowland Hill C (2018). Imaging assessment of acute ischaemic stroke: a review of radiological methods. Br J Radiol.

[4] Kleindorfer DO, Towfighi A, Chaturvedi S (2021). 2021 Guideline for the Prevention of Stroke in Patients With Stroke and Transient Ischemic Attack: a guideline from the American Heart Association/American Stroke Association. Stroke.

[5] Baumgartner RW, Bogousslavsky J, Caso V, Paciaroni M (2005). Handbook on Cerebral Artery Dissection. https://www.ncbi.nlm.nih.gov/pmc/articles/PMC8139770/pdf/0162.pdf.

[6] Bratu IF, Ribigan AC, Stefan D, Davidoiu CR, Badea RS, Antochi FA (2020). Internal carotid artery dissection - a case for antithrombotic therapy in the era of (minimally) invasive procedures. Maedica (Bucur).

[7] Biller J, Sacco RL, Albuquerque FC (2014). Cervical arterial dissections and association with cervical manipulative therapy: a statement for healthcare professionals from the american heart association/american stroke association. Stroke.

[8] Morel A, Naggara O, Touzé E, Raymond J, Mas JL, Meder JF, Oppenheim C (2012). Mechanism of ischemic infarct in spontaneous cervical artery dissection. Stroke.

[9] Benninger DH, Georgiadis D, Kremer C, Studer A, Nedeltchev K, Baumgartner RW (2004). Mechanism of ischemic infarct in spontaneous carotid dissection. Stroke.

[10] Zinkstok SM, Vergouwen MD, Engelter ST (2011). Safety and functional outcome of thrombolysis in dissection-related ischemic stroke: a meta-analysis of individual patient data. Stroke.

[11] Menon R, Kerry S, Norris JW, Markus HS (2008). Treatment of cervical artery dissection: a systematic review and meta-analysis. J Neurol Neurosurg Psychiatry.

[12] Georgiadis D, Arnold M, von Buedingen HC (2009). Aspirin vs anticoagulation in carotid artery dissection: a study of 298 patients. Neurology.

[13] Markus HS, Levi C, King A, Madigan J, Norris J (2019). Antiplatelet therapy vs anticoagulation therapy in cervical artery dissection: the cervical artery dissection in stroke study (CADISS) randomized clinical trial final results. JAMA Neurol.

[14] Engelter ST, Traenka C, Gensicke H (2021). Aspirin versus anticoagulation in cervical artery dissection (TREAT-CAD): an open-label, randomised, non-inferiority trial. Lancet Neurol.

[15] Hagrass AI, Almaghary BK, Mostafa MA (2022). Antiplatelets versus anticoagulation in cervical artery dissection: a systematic review and meta-analysis of 2064 patients. Drugs R D.

[16] Johnston SC, Easton JD, Farrant M (2018). Clopidogrel and aspirin in acute ischemic stroke and high-risk TIA. N Engl J Med.

[17] Wang Y, Wang Y, Zhao X (2013). Clopidogrel with aspirin in acute minor stroke or transient ischemic attack. N Engl J Med.

[18] Albay CE, Leyson FG, Cheng FC (2020). Dual versus mono antiplatelet therapy for acute non-cardio embolic ischemic stroke or transient ischemic attack, an efficacy and safety analysis - updated meta-analysis. BMC Neurol.

[19] Xianjun H, Zhiming Z (2013). A systematic review of endovascular management of internal carotid artery dissections. Interv Neurol.

[20] Kremer C, Mosso M, Georgiadis D, Stöckli E, Benninger D, Arnold M, Baumgartner RW (2003). Carotid dissection with permanent and transient occlusion or severe stenosis: long-term outcome. Neurology.

